# Inadvertent Epidural and Intravenous Line Swap: A Case Report

**DOI:** 10.7759/cureus.36698

**Published:** 2023-03-26

**Authors:** Carolina S Dias, Carla I Ferreira, Rui V Torres, Juliana L Cruz

**Affiliations:** 1 Anesthesiology, Centro Hospitalar Lisboa Central, Lisbon, PRT; 2 Anesthesiology, Hospital de Braga, Braga, PRT

**Keywords:** morbidity and mortality, anesthesia-related human error, local anesthetic systemic toxicity, inadvertent epidural administration, administration errors

## Abstract

Administration of medication via the wrong administration route has the potential for serious morbidity and mortality. Regrettably, because of the ethical implications in such situations, most of our knowledge comes from case reports. This paper reports on the accidental misconnection of intravenous acetaminophen to an epidural line and of the patient-controlled epidural analgesia (PCEA) pump to intravenous access, as a result of patient error.

A male patient aged 60-65 years, 80 kg, American Society of Anesthesiologists (ASA) physical status III presented for unilateral total knee arthroplasty under a combined spinal-epidural anaesthesia technique. For postoperative analgesia, a multimodal analgesia regimen including acetaminophen, in combination with a PCEA pump, was selected.

During the night, the patient disconnected and reconnected the drug administration lines, resulting in an epidural/intravenous misconnection. After six unsupervised hours, a total of 114 mg of ropivacaine was administered intravenously and the acetaminophen vial, at this time connected to the epidural catheter, was found empty. A full physical examination by the on-call anaesthesiologist showed no abnormal findings and the nursing staff and patient were instructed on signs to look out for and how to monitor for complications.

This case highlights the risks associated with intravenous/epidural line misconnection, as well as the impactful variable the patient represents when admitted to a lower vigilance infirmary. This makes it evident that more safety developments are needed to ensure the utmost quality of care is provided to all patients.

## Introduction

Inadvertent administration of any medication via the wrong administration route has the potential for serious morbidity and mortality [1-6]. Given the potential complications associated with such errors, and the absence of procedural guidelines or specific treatment in the event they happen [2-6], prevention should be the main strategy [1,3,4,6,7]. Regrettably, because of the ethical implications in such situations, most of our knowledge comes from case reports [2,4,6,7]. As such, communicating these errors and any clinical repercussions associated with them is of extreme importance for professionals who are faced with similar situations in their medical practice [2].

The frequency of inadvertent injection of drugs in the epidural space is likely underestimated [1,2,5,6] and underreported, with neurological complications such as paraplegia, urinary incontinence, or sensory alterations [2,4-6] being our main concern. This paper reports on the accidental misconnection of intravenous acetaminophen to an epidural line and of the patient-controlled epidural analgesia (PCEA) pump to an intravenous access. Despite epidural/intravenous line confusion being a previously reported administration error [5], to our knowledge it has never been reported as a direct result of patient error. In this instance, in addition to side effects regarding wrongful epidural administration, local anaesthetic systemic toxicity (LAST) was also a potential concern.

## Case presentation

A male patient aged 60-65 years, 80 kg, American Society of Anesthesiologists (ASA) physical status III, with no known allergies and with a history of diabetes mellitus, essential hypertension, obesity, dyslipidaemia, chronic renal disease, and alcoholic liver disease, presented for unilateral total knee arthroplasty. Given the expected postoperative pain score [8], a combined spinal-epidural anaesthesia technique was selected for intraoperative anaesthesia and postoperative analgesia. The epidural catheter placement was performed without any complications. For postoperative analgesia, a multimodal analgesia regimen in combination with a PCEA pump was selected. Regular administration of intravenous acetaminophen every 6 hours was prescribed with tramadol 100 mg as rescue analgesia. Additionally, the selected PCEA pump settings accounted for regular 6 mL boluses every 4 hours as well as hourly patient-controlled 4 mL boluses of a fentanyl 2 µg/mL and ropivacaine 1.5 mg/mL mixture.

As the patient reported uncontrolled pain on the first postoperative day, PCEA settings were adjusted, by increasing the frequency of fixed boluses to every 2 hours and reducing the lockout interval for PCEA rescue boluses to every 30 minutes, without background infusion.

On the second night after the surgery, the prescribed intravenous acetaminophen was connected at midnight by the nursing staff, who concurrently confirmed adequate PCEA performance and connection. The following morning, at 6 am, the patient was found asleep with the intravenous line connected to the epidural catheter and the PCEA pump connected to the intravenous line. When questioned, the patient mentioned waking up to use the washroom facilities and personally disconnecting and reconnecting the lines himself but was not able to specify at what time this event had taken place. Both lines were immediately disconnected by the nursing staff and the anaesthesiology department was contacted and informed of the situation. A maximum total of 114 mg ropivacaine and 152 µg fentanyl were estimated to have been administered intravenously over the 6 hours the patient was unsupervised, accounting for fixed and patient-controlled boluses. The acetaminophen vial was empty when the connection error was detected. Given the situation, the on-call anaesthesiologist performed a full physical examination, with emphasis on neurologic and cardiac adverse signs and symptoms, as well as full blood work and 12-lead electrocardiogram, with no abnormal findings at that time. Due to the risk of delayed clinical presentation, the patient was initially monitored every 2 hours in a high-dependency-care unit, where he remained for the first 24 hours, before returning to the orthopaedics' infirmary. The nursing staff and the patient were instructed on what warning signs to be aware of and monitor closely for, with particular emphasis on arterial blood pressure variations, electrocardiographic abnormalities and neurological clinical symptoms (such as perioral numbness, metallic taste or mental status changes) [7].

Taking into consideration the patient, the potential complications, and the still poorly controlled pain, the PCEA pump was no longer considered an adequate analgesia regimen and was replaced with regular epidural boluses of 12 mg ropivacaine and 10 µg sufentanil solely administered by the nursing staff.

No side effects were reported for the duration of the hospitalization and on the fifth postoperative day the patient was considered fit for discharge. Warning signs and symptoms to look out for were explained once more and the need for medical re-evaluation in the event of their occurrence was reinforced.

## Discussion

This case report brings to light three main concerns regarding an event of wrongful drug administration both intravenously and in the epidural space: the potential side-effects associated with either drug, as well as the fact that despite all safety measures adopted by health professionals to prevent such occurrences, the patient himself remains a variable out of our control.

Acetaminophen is a frequently used intravenous analgesic [3,9] favoured for its safety profile [1,9]. However, despite its frequent use and ready availability [3,9], no safety trials were run regarding its epidural administration [3]. Currently, there are some case report descriptions of erroneous epidural administration of acetaminophen without complications or development of neurotoxicity symptoms [1,3,6,9]. The majority of those report back on syringe or ampule swaps by healthcare professionals [1-6]. However, in this case, the acetaminophen’s low-pressure intravenous system was passively connected to the epidural catheter. After some consideration, the formulated hypothesis was that, as the epidural system is, by nature, a high-pressure one, there might not have been any administration of the actual drug. This would be the most likely scenario given that the intravenous systems would not have the power to overcome such pressures without active syringe administration, which was not the case. As such, the acetaminophen vial must have run its course while still connected intravenously and before the line swap took place. This could also account for the lack of immediate or latent neurotoxicity symptoms demonstrated by the patient, even though none of the available case reports describe any such manifestations [1]. Alternatively, the acetaminophen might have slowly been administered over the course of 6 hours and, as such, might have had its effects, both adverse and therapeutic, attenuated [5,6]. Irrespective of which hypothesis is true, no complications or side effects were developed by the patient, who remains in good health to this day.

Additionally, ropivacaine, a frequently used local anaesthetic [10], was also freely administered intravenously. Even though there is not extensive research on most wrongful drug administrations [5,6], this is a well-studied one, as it is also a potential complication of peripheral nerve blocks or neuraxial anaesthesia [10]. LAST is a potentially deadly complication of local anaesthetic intravenous administrations that courses with neurological and cardiac symptoms which must be readily recognized in order to implement general symptomatic treatment and take resuscitation measures when necessary [7]. Given its mortality most hospitals have specific procedure protocols in place [7] when such events are identified. Another preventive measure implemented when using local anaesthetics is the calculation of the maximum recommended dose for each local anaesthetic [7,10]. This will ensure that even if intravenous administration does occur, the chances of serious side effects are reduced [7] as long as the maximum safe dosages are complied with. For ropivacaine a 3 mg per kg [7,10] maximum safe dosage is considered, when administered perineurally, which for the patient would mean a maximum dose of 240 mg of ropivacaine. Considering the scenario described by the patient and nursing staff, he was left unsupervised for a maximum of 6 hours. Assuming worst case scenario and that the intravenous line and epidural catheter were misconnected for the full period, a maximum of four fixed 6 mL boluses and 13 patient-controlled 4 mL boluses were given. This puts the maximum possible amount of ropivacaine administered at 114 mg. However, even though this estimated dosage is under the considered maximum safe perineural dose for ropivacaine, it does not apply for intravascular administrations, as this is never the intended use of ropivacaine. With this in mind, any cardiological and neurological side effects were also excluded, for maximum patient safety. Additionally, the patient was informed of the potential risks and of any symptoms he might experience in the future. The nursing staff was equally advised of such symptoms and given specific instructions to monitor this patient closely. Any new development was to be promptly communicated to the anaesthesiologist on call.

As there is no specific antidote available to us for when intravenous local anaesthetic administration occurs [7], close monitoring of vital signs and any new symptom development is paramount. In this case, given the asymptomatic and stable status of the patient, it was considered safe to monitor the patient in a regular orthopaedics’ infirmary environment, after a 24-hour period in a high-dependency-care unit. However, had there been any alarm signs, transfer to a level 1 intensive care unit bed and further complementary exams would have been warranted.

Safety measures and protocols have been and are still being developed to prevent erroneous drug administration. A review of the literature revealed that, even though a great amount of effort is being put into reducing inadvertent drug administration [5], the patient has yet to be considered a modifiable variable. The lack of control over patients makes them a potentially dangerous source of interference when it comes to their own safety. In this case, during the period of least vigilance in a regular infirmary, a patient unknowingly put his own wellbeing at risk when simple measures such as patient education and closer monitoring [6] of such devices could have prevented this occurrence. This highlights the importance of discussing analgesic options with patients before considering implementing non-conventional analgesia devices. Proper clarification and adequate information may be enough to ensure that most patients know not to interfere with intravenous or epidural accesses. However, not all patients can achieve this level of understanding, and in those cases, in which this patient would have to be included, other safety measures would have to be considered. Ideally, different connection latches should be used for both the intravenous and epidural lines [3,5,6] as this would ensure that even inadvertently, no one could misconnect them. For many years now, the small-bore connector most often used in the medical device field has been the universal Luer connector [11]. Unfortunately, the simplicity of the Luer connector allows the tube of the device for one delivery system to be connected to an unrelated system with a different intended use [11]. This is what, in some circumstances, results in unintended drugs being delivered through the wrong route of administration [11] and a small modification at this level could be a simple way to safeguard our patients against this potentially dangerous event. The use of this modified connector, in lieu of Luer connectors, would prevent medical devices meant for neuraxial administration from connecting to devices used for any other intended route and vice-versa [11]. This would ensure that only drugs prepared and available in epidural-specific systems could be administered that way, and it would similarly ensure that the drugs available in such systems would not be administered intravenously, as the latch mechanisms would be incompatible. It is thus evident that, had a mechanism such as this been in use at the time of this incident, the entire event could have been avoided completely.

Some countries have begun approving laws mandating the use of physically incompatible tubing connectors for different routes of clinical application [12]. The increasing implementation of this safety measure should be an important contributor towards reducing tubing misconnections, as well as help identify other patient safety concerns that may arise [12]. This, in addition to the continued occurrence of clinical cases similar to this one, reinforces the need for more countries to follow suit.

## Conclusions

Ultimately, as a result of the critical incident report filed describing this situation, patient education and nursing staff training were intensified, with a specific focus on non-conventional analgesia techniques. Additionally, anaesthesiologists were encouraged to approach possible options for post-operative pain control during the pre-operative anaesthesia appointment, in order to clarify any potential doubts or misconceptions.

However, regardless of the measures undertaken, this case report makes evident how crucial the implementation of a different product line in this area is, in order to ensure that the utmost quality of care is provided to all patients. With this in mind, an official product request was drafted and sent up for administrative approval, initiating the process towards the improvement of patient care. Hopefully, in time, case reports of this nature will no longer be relevant, as these will be the driving force towards ensuring the availability of appropriate and distinct intravenous and epidural connection locks in every hospital.
